# Early left atrial reverse remodelling in patients with hypertrophic obstructive cardiomyopathy receiving transapical beating-heart septal myectomy

**DOI:** 10.1093/icvts/ivae145

**Published:** 2024-08-09

**Authors:** Shirui Lu, Jun Zhang, Ying Zhu, Wei Zhou, Xueqing Cheng, Hui Wang, Yue Chen, Xiang Wei, Yani Liu

**Affiliations:** Department of Medical Ultrasound, Tongji Hospital, Tongji Medical College, Huazhong University of Science and Technology, Wuhan, Hubei, People’s Republic of China; Department of Medical Ultrasound, Tongji Hospital, Tongji Medical College, Huazhong University of Science and Technology, Wuhan, Hubei, People’s Republic of China; Department of Medical Ultrasound, Tongji Hospital, Tongji Medical College, Huazhong University of Science and Technology, Wuhan, Hubei, People’s Republic of China; Department of Medical Ultrasound, Tongji Hospital, Tongji Medical College, Huazhong University of Science and Technology, Wuhan, Hubei, People’s Republic of China; Department of Medical Ultrasound, Tongji Hospital, Tongji Medical College, Huazhong University of Science and Technology, Wuhan, Hubei, People’s Republic of China; Department of Medical Ultrasound, Tongji Hospital, Tongji Medical College, Huazhong University of Science and Technology, Wuhan, Hubei, People’s Republic of China; Department of Cardiovascular Surgery, Tongji Hospital, Tongji Medical College, Huazhong University of Science and Technology, Wuhan, Hube, People’s Republic of China; Department of Cardiovascular Surgery, Tongji Hospital, Tongji Medical College, Huazhong University of Science and Technology, Wuhan, Hube, People’s Republic of China; Department of Medical Ultrasound, Tongji Hospital, Tongji Medical College, Huazhong University of Science and Technology, Wuhan, Hubei, People’s Republic of China

**Keywords:** Hypertrophic cardiomyopathy, Transapical beating-heart septal myectomy, Left ventricular diastolic function, Left atrial reverse remodelling

## Abstract

**OBJECTIVES:**

This study aims to investigate the short-term effects of transapical beating-heart septal myectomy (TA-BSM) on left atrial (LA) anatomy and function and its association with clinical indicators in patients with hypertrophic obstructive cardiomyopathy (HOCM).

**METHODS:**

A total of 105 HOCM patients who received TA-BSM were included. Clinical and comprehensive echocardiographic data were obtained before surgery, at discharge, and 3 months after myectomy. LA reverse remodelling was defined as LA maximum volume index (LAVI) ≤34 ml/m^2^ and a change of ≥10%.

**RESULTS:**

At 3 months after TA-BSM, New York Heart Association (NYHA) functional class and 6-min walking test were significantly improved, N-terminal pro–B-type natriuretic peptide (NT-proBNP) decreased, left ventricular outflow tract (LVOT) peak gradient and mitral regurgitation were significantly reduced. LAVI decreased in 76%, with a median change of 20%, and the criteria for LA reverse remodelling were met in 48%. LA strain parameters were improved at 3 months after TA-BSM. Moreover, left ventricular (LV) diastolic function was significantly improved, but LV global longitudinal strain was not significantly changed at 3 months after operation. Improvement in LVOT peak gradient, LAVI, LA reservoir strain (LASr) and conduit strain (LAScd) were associated with reduction in NT-proBNP.

**CONCLUSIONS:**

Along with effectively relieving the obstruction of the LVOT and mitral regurgitation, TA-BSM could significantly improve LA size and function during the short-term follow-up for HOCM patients. The indicators of LA reverse remodelling were associated with reduction in a biomarker of myocardial wall stress, indicating the early recovery of LV relaxation and clinical status for patients.

## Graphical Abstract

**Figure:**
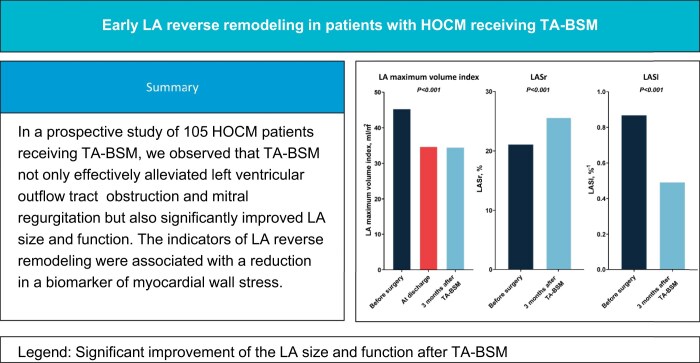

## INTRODUCTION

Hypertrophic cardiomyopathy (HCM) is an inherited heart disease with left ventricular outflow tract obstruction (LVOTO) in 70% of patients [1]. HCM patients, particularly those with hypertrophic obstructive cardiomyopathy (HOCM), often experience left atrial (LA) remodelling and left ventricular (LV) diastolic dysfunction, which increase the risk of sudden cardiac death, atrial fibrillation, stroke and heart failure [2]. Recent research has highlighted the dynamic role of the LA in overall cardiac performance during treatment [3]. Nuzzi *et al*. [4] investigated LA reverse remodelling in dilated cardiomyopathy, defining it as LA maximum volume index (LAVI) ≤34 ml/m^2^ with >10% change, and found that patients with such remodelling had lower risks of adverse outcomes at 1-year follow-up. These studies indicated that the LA reverse remodelling could be a marker of the positive treatment and has a close relationship with the outcome.

Surgical septal myectomy remains the preferred treatment for most HOCM patients [5]. Transapical beating-heart septal myectomy (TA-BSM) is a newer, minimally invasive approach for septal reduction in HOCM, offering real-time haemodynamic assessment [6]. In our first group of 47 HOCM patients who underwent TA-BSM procedures, 89.4% achieved procedural success within 3 months [7]. While early outcomes of TA-BSM may not surpass those of conventional transaortic myectomy in terms of surgical risk or LVOTO relief, its avoidance of cardiopulmonary bypass and minimally invasive nature support faster patient recovery. This study aims to explore TA-BSM’s early effects on cardiac structure and function, particularly focusing on early changes in LA size and function and their correlation with clinical indicators.

## METHODS

This was a single-centre, single-arm, first-in-human registry study. The study followed ethical standards and received approval from the Ethics Committee of Tongji Medical College. It has been registered on ClinicalTrials.gov (NCT05332691).

### Study population

From April 2022 to July 2023, 105 HOCM patients undergoing TA-BSM were included if they were aged ≥12 years with septal thickness ≥15 mm, left ventricular outflow tract (LVOT) gradient >50 mmHg (rest/provoked), New York Heart Association (NYHA) class II+, and refractory symptoms or drug intolerance. Exclusions were native valvular disease, coronary artery disease, LV ejection fraction (LVEF) <40%, pregnancy, life expectancy <1 year, poor compliance, or other conditions deemed unsuitable by the medical team. The apical puncture site is located accurately using finger palpation under transthoracic echocardiogrpahy. Then an 18-G needle punctures the apex, and a 0.035-inch wire is inserted into the LV. After dilating the puncture site, a myectomy device is inserted under transoesophageal echocardiography guidance. It precisely navigates the device entering into LVOT and contacting the target septum without interfering with the mitral valve or aortic valve. The more detailed procedures can be referred to our previously study published on JACC [7].

### Echocardiography

Comprehensive echocardiograms were performed on all patients before surgery, at discharge, and at 3 months after TA-BSM. Ultrasound used GE Vivid E95 with an M5Sc transducer. Parameters followed American Society of Echocardiography guidelines [8]. Mitral valve distance to septum were measured at late-systole [9]. Systolic anterior motion (SAM) were categorized by SAM-septal distance and leaflet-septal contact duration [10]. Mitral regurgitation (MR) grades (0 to 4+) were assessed [11]. Peak LVOT gradient was measured in apical views; provocation tests were used for patients with resting gradient under 50 mmHg [12]. LA reverse remodelling was defined as LAVI ≦34 ml/m^2^ and a change of ≧10% [4].

### Statistical analysis

Continuous variables were normality verified using Kolmogorov–Smirnov test. Statistical tests accounted for paired data comparing pre- and postintervention characteristics. For categorical data, McNemar test replaced chi-squared. Paired Student’s *t*-test compared normally distributed continuous data, and repeated-measures ANOVA for multiple groups. Wilcoxon’s signed rank test compared non-normally distributed continuous data between two groups, and Friedman test for multiple groups. Log2-transformed N-terminal pro-B-type natriuretic peptide [NT-proBNP] and high-sensitivity cardiac troponin I [hs-cTnI] data underwent simple linear regression with echocardiographic parameters. *P* < 0.05 indicated significance. SPSS Version 23.0 (IBM Corporation, Armonk, NY, USA) performed all analyses.

## RESULTS

### Patient characteristics

The baseline characteristics of 105 patients with HOCM who underwent the TA-BSM are listed in Table 1. The median (IQR) of age was 48 (34, 59) years, 95 patients were adults and 10 were paediatric, and 68 (64.8) were men.

**Table 1:** Baseline clinical characteristics

Characteristic	No. (%)
Clinical characteristic	
Male	68 (65)
Female	37 (35)
Age, y	48 (12, 77)
BSA, m^2^	1.78 ± 0.20
Clinical symptoms	
Shortness of breath	56 (53)
Chest pain	52 (50)
Chest tightness	53 (51)
Syncope	14 (13)
Palpitations	51 (49)
Dizziness	15 (14)
Amaurosis	18 (17)
Hypodynamic	8 (8)
NYHA functional classification	
I	1 (1)
II	48 (46)
III	53 (50)
IV	3 (3)
Family history of HCM	21 (20)
Comorbidities	
Hypertension	31 (30)
Diabetes mellitus	4 (4)
Kidney disease	8 (8)
Coronary artery disease	12 (11)
Medications	
β-Blocker	59 (56)
Calcium channel blocker	31 (30)

BSA: body surface area; HCM: hypertrophic cardiomyopathy; NYHA: New York Heart Association.

### Follow-up clinical outcomes

**Figure 1:**
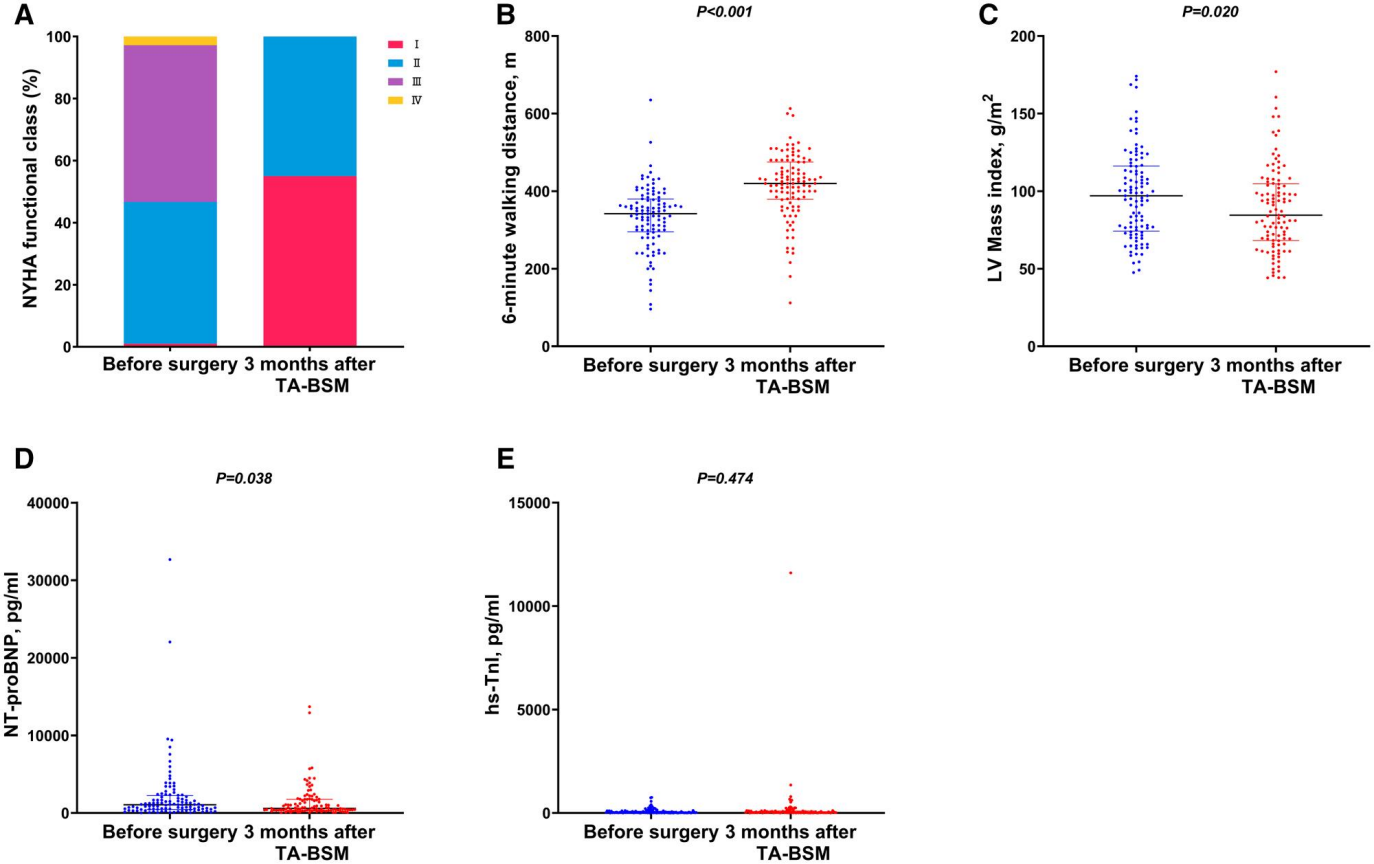
Clinical outcomes at 3 months after TA-BSM. (**A**) NYHA functional class; (**B**) 6-min walking distance; (**C**) LV mass index; (**D**) NT-proBNP; (**E**) hs-cTnI. NYHA: New York Heart Association; LV: left ventricular; NT-proBNP: N-terminal pro-B-type natriuretic peptide; hs-cTnI: high-sensitivity cardiac troponin I.

Device success was achieved in 103 of the 105 patients, except for 2 patients who were converted to sternotomy. During the short-term follow-up, 64 patients developed left bundle branch block. The representative clinical outcomes are shown in Table 2. At 3 months after TA-BSM, % of patients with NYHA III/IV symptoms decreased from 53% to 1%. All 105 patients showed the disappearance or significant alleviation of their symptoms and improvements in exertional capacity. There was a significant increase in median (IQR) 6-min walking distance from 342 (298, 379) m to 420 (380, 475) m (*P* < 0.001). The LV mass index was significantly lower than that before surgery (97.52 ± 28.55 g/m^2^ vs 88.42 ± 27.50 g/m^2^, *P* = 0.020). Compared with baseline, the level of NT-proBNP was significantly lower [1062 (476, 2153) pg/ml vs 585 (337, 1759) pg/ml, *P* = 0.038], while the level of hs-cTnI showed no significant change [23 (12, 85) pg/ml vs 28 (14, 91) pg/ml, *P* = 0.474] (Table 2, Fig. 1).

**Table 2:** Clinical outcomes after myectomy 1–3 months

Variable	Baseline (*n* = 105)	3 months after myectomy (*n* = 105)	*P*-value
NYHA functional classification			<0.001
I	1 (1)	55 (52)	
II	48 (46)	49 (47)	
III	53 (50)	0 (0)	
IV	3 (3)	1 (1)	
III or IV	56 (53)	1 (1)	
Clinical symptoms			
Shortness of breath	56 (53)	10 (10)	<0.001
Chest pain	52 (50)	3 (3)	<0.001
Chest tightness	53 (51)	12 (11)	<0.001
Syncope	14 (13)	0 (0)	<0.001
Palpitations	51 (49)	3 (3)	<0.001
Dizziness	15 (14)	2 (2)	0.002
Amaurosi	18 (17)	3 (3)	<0.001
Hypodynamic	8 (8)	5 (5)	0.567
6-min walking test (m)	342 (298, 379)	420 (380, 475)	<0.001
Laboratory examination			
NT-proBNP (pg/ml)	1062 (476, 2153)	585 (337, 1759)	0.038
cTnI (pg/ml)	23 (12, 85)	28 (14, 91)	0.474
CMR			
LV mass index (g/m^2^)	97.52 ± 28.55	88.42 ± 27.50	0.020

CMR: cardiac magnetic resonance; hs-cTnI: high-sensitivity cardiac troponin I; LV: left ventricular; NT-proBNP: N-terminal pro-B-type natriuretic peptide; NYHA: New York Heart Association.

### Follow-up echocardiographic parameters

**Figure 2:**
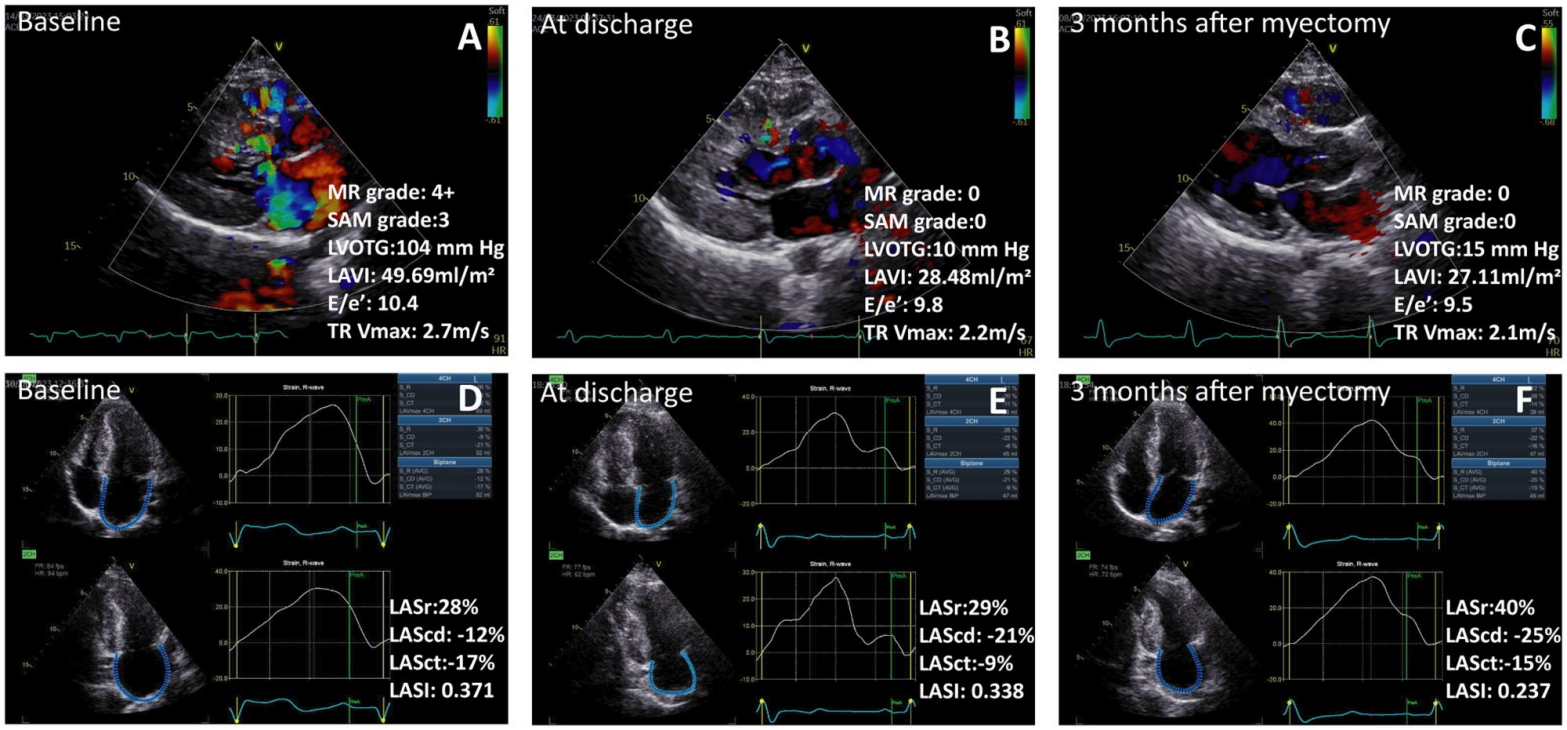
Treatment with transapical beating-heart septal myectomy led to significant decreased thickness of the interventricular septum, relieving the obstruction of the LVOT and MR grade, improved left atrial size and function, improve LV diastolic function during the short-term follow-up for hypertrophic obstructive cardiomyopathy patients. MR: mitral regurgitation; SAM: systolic anterior motion; LVOTG: left ventricular outflow tract grade; LAVI: left atrial maximum volume index; E/e′: early mitral inflow velocity/the average of septal and lateral mitral annular early diastolic velocity; TR Vmax: tricuspid regurgitation maximum velocity; LASr: left atrial reservoir strain; LAScd: left atrial conduit strain; LASct: left atrial contraction strain; LASI: left atrial stiffness index.

The comparison of echocardiographic parameters among all patients before surgery, at discharge, and three months after surgery is shown in Fig. 2 and Table 3. There was a significant reduction in LVOT peak gradient [69 (37, 97) mmHg vs 13 (9, 18) mmHg, *P* < 0.001], and a LVOT peak gradient of <30 mmHg was achieved in 99 of 105 patients at 3 months after TA-BSM. Compared with baseline, there was a significant reduction in the proportion of mitral regurgitation ≥ 2+ [88(84%) vs 9(9%), *P* < 0.001] and SAM grade ≥ 2 [91(87%) vs 6(6%), *P* < 0.001] at 3 months after operation. The LVOT diameter [13 (11, 16) mm vs 17 (16, 19) mm, *P* < 0.001] and closest distance between coaptation point of mitral valve and ventricular septum [6.14 (4.62, 9.00) mm vs 17.00 (14.00, 20.70) mm, *P* < 0.001] was increased at 3 months after TA-BSM. LV dimensional parameters showed improvements at 3 months after TA-BSM, including decreased thickness of the interventricular septum at the end-diastolic, and enlarged LV end-diastolic volume.

**Table 3:** Echocardiographic parameters and follow-up outcomes

Parameters	Baseline (*n* = 105)	At discharge (*n* = 105)	3 months after myectomy (*n* = 105)	*P*-value
Anterior IVS thickness (base) (mm)	20.9 (18.0, 25.0)	13.0 (12.0, 15.0)^*^	13.0 (11.5, 14.0)^△^	<0.001
Anterior IVS thickness (mid) (mm)	19.9 (15.0, 25.4)	14.2 (12.2, 18.0)^*^	15.0 (11.0, 17.2)^△^	<0.001
Posterior IVS thickness (base) (mm)	17.1 (15.0, 21.0)	13.0 (12.0, 16.8)^*^	13.0 (11.1, 16.0)^△^	<0.001
Posterior IVS thickness (mid) (mm)	20.0 (16.0, 26.0)	16.0 (12.8, 19.5)^*^	15.0 (12.0, 18.0)^△^	<0.001
Maximum IVS thickness (mm)	23.0 (19.0, 28.0)	17.0 (15.0, 20.1)^*^	16.0 (13.8, 20.0)^△^	<0.001
LV posterior wall (mm)	9.0 (9.0, 11.0)	12.0 (9.0, 16.0)^*^	12.9 (10.0, 18.0)^△^	<0.001
LVOT diameter (mm)	13 (11, 16)	16 (14, 18)^*^	17 (16, 19)^△^^#^	<0.001
LVOT peak gradient (mmHg)	69 (37, 97)	15 (10, 19)^*^	13 (9, 18)^△^	<0.001
<30 mmHg	21 (20)	97 (92)^*^	99 (94.3)^△^	<0.001
≥30 mmHg	84 (80)	8 (8)^*^	6 (5.7)^△^	<0.001
MV to IVS (mm)	6.14 (4.62, 9.00)	15.28 (12.32, 18.00)^*^	17.00 (14.00, 20.70)^△^^#^	<0.001
SAM grade				<0.001^*^^△^
0	5 (5)	42 (40)	48 (45.7)	
1	9 (9)	57 (54)	51 (48.6)	
2	24 (23)	5 (5)	6 (5.7)	
3	36 (34)	1 (1)	0 (0.0)	
4	31 (30)	0 (0)	0 (0.0)	
MR grade				<0.001^*^^△^^#^
0	0 (0)	19 (18)	34 (32.4)	
1+	17 (16)	70 (67)	62 (59.0)	
2+	23 (22)	12 (11)	8 (7.7)	
3+	36 (34)	3 (3)	0 (0.0)	
4+	29 (28)	1 (1)	1 (0.9)	
MR area/LA area (%)	32 (23, 41)	11 (7, 16)^*^	8 (6, 13)^△^^#^	<0.001
LVD (mm)	44.0 (40.0, 47.0)	48.0 (47.0, 51.0)^*^	50.0 (46.0, 52.0)^△^	<0.001
LVEDV (ml)	83.0 (73.6, 100.0)	91.0 (82.0, 103.0)^*^	104.0 (90.0, 116.0)^△^^#^	<0.001
LVESV (ml)	27.0 (23.0, 32.4)	30.0 (25.0, 36.0)^*^	35.0 (26.0, 46.0)^△^^#^	<0.001
LVEF (%)	67.0 (62.0, 74.0)	68.0 (62.0, 72.0)	66.0 (60.0, 70.0)^△^	0.010
LV longitudinal strain (%)	−13.0 (−15.4, −9.2)	NA	−12.9 (−15.0, −10.0)	0.797
LAD (mm)	42.0 (39.8, 46.0)	38.0 (35.0, 42.0)^*^	37.0 (34.0, 40.0)^△^	<0.001
LAVI（ml/m^2^）	43.5 (36.1, 55.4)	NA	33.2 (27.8, 39.6)^△^	<0.001
LAEDV (ml)	80.0 (64.1, 92.0)	57.0 (44.0, 75.0)^*^	59.0 (49.0, 72.0)^△^	<0.001
LAESV (ml)	39.8 (29.0, 52.0)	27.0 (20.0, 37.0)^*^	25.0 (19.7, 31.2)^△^	<0.001
LAEF (%)	49.1 ± 10.7	52.2 ± 8.5^*^	55.7 ± 8.0^△^^#^	<0.001
LASr (%)	20.0 (16.0, 25.0)	NA	25.0 (21.0, 31.0)^△^	<0.001
LAScd (%)	−10.0 (−13.0, −7.0)	NA	−12.0 (−15.00, −9.0)^△^	0.012
LASct (%)	−10.0 (−13.0, −7.0)	NA	−13.0 (−16.0, −10.0)^△^	<0.001
E/e′ (average)	13.6 (10.8, 17.3)	12.1 (9.2, 14.7)^*^	10.9 (9.1, 13.3)^△^	<0.001
Ar-A duration (ms)	34.00 (16.00, 48.00)	13.50 (−0.50, 29.75)^*^	10.00 (−8.00, 20.00)^△^^#^	<0.001
TR Vmax (m/s)	2.5 (2.2, 2.8)	2.1 (1.9, 2.4)^*^	2.2 (1.8, 2.4)^△^	<0.001
LA stiffness index (%^−1^)	0.65 (0.48, 1.05)	NA	0.42 (0.30, 0.60)^△^	<0.001

*△ *P* < 0.05 versus baseline,

# *P* < 0.05 versus at discharge.

Ar-A duration: the time difference between duration of pulmonary vein flow and mitral inflow during atrial contraction; E/e′: early mitral inflow velocity/the average of septal and lateral mitral annular early diastolic velocity; IVS: interventricular septum; LA: left atrial; LAD: left atrial end-systolic dimension; LAEDV: left atrial end-diastolic volume; LAEF: left atrial ejection fraction; LAESV: left atrial end-systolic volume; LAScd: left atrial conduit strain; LASct: left atrial contraction strain; LASr: left atrial reservoir strain; LAVI: left atrial maximum volume index; LV: left ventricular; LVD: left ventricular end-diastolic dimension; LVEDV: left ventricular end-diastolic volume; LVEF: left ventricular ejection fraction; LVESV: left ventricular end-systolic volume; LVOT: left ventricular outflow tract; MR: mitral regurgitation; MV to IVS: closest distance between coaptation point of mitral valve and ventricular septum was measured at late-systolic; SAM: systolic anterior motion; TR Vmax: tricuspid regurgitation maximum velocity.

**Figure 3:**
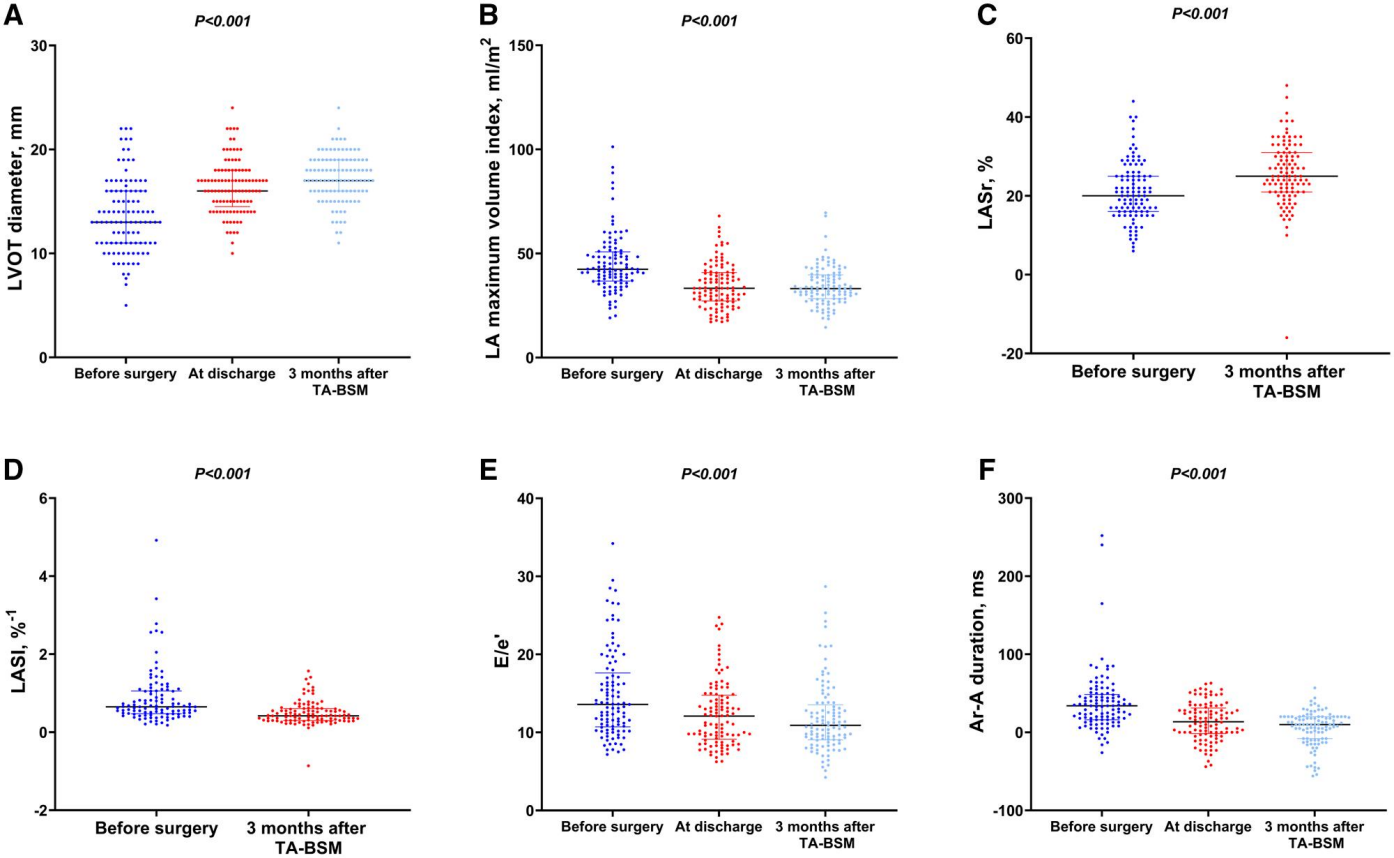
Echocardiographic parameters and follow-up outcomes. (**A**) LVOT diameter; (**B**) LA maximum volume index; (**C**) LASr; (**D**) LASI; (**E**): E/e′; (**F**): Ar-A duration. LVOT: left ventricular outflow tract; LA: left atrial; LASr: left atrial reservoir strain; LASI: left atrial stiffness index; E/e′: early mitral inflow velocity/the average of septal and lateral mitral annular early diastolic velocity; Ar-A duration: the time difference between duration of pulmonary vein flow and mitral inflow during atrial contraction.

At 3 months after myectomy, LAVI decreased in 80 of 105 (76%) patients, with a median change of 20%, and the criteria for LA reverse remodelling were met in 50 of 105 (48%) patients. Moreover, there was significant improvement of LV diastolic function, including decreased early mitral inflow velocity and the average of septal and lateral mitral annular early diastolic velocity (E/e′) [13.6 (10.8, 17.3) vs 10.9 (9.1, 13.3), *P* < 0.001], the time difference between duration of pulmonary vein flow and mitral inflow during atrial contraction (Ar-A) [34.00 (16.00, 48.00) ms vs 10.00 (−8.00, 20.00) ms, *P* < 0.001], tricuspid regurgitation maximum velocity [2.5 (2.2, 2.8) m/s vs 2.2 (1.8, 2.4) m/s, *P* < 0.001] and LA stiffness index [0.65 (0.48, 1.05)%^−1^ vs 0.42 (0.30, 0.60)%^−1^, *P* < 0.001] (Fig. 3). There was also significant improvement of LA strains, including LA reservoir strain (LASr) [20.0 (16.0, 25.0)% vs 25.0 (21.0, 31.0)%, *P* < 0.001], LA conduit strain (LAScd) [−10.0 (−13.0, −7.0)% vs −12.0 (−15.00, −9.0)%, *P* < 0.001] and LA contraction strain (LASct) [−10.0 (−13.0, −7.0)% vs −13.0 (−16.0, −10.0)%, *P* < 0.001], while LV global longitudinal strain (GLS) [−13.0 (−15.4, −9.2)% vs −12.9 (−15.0, −10.0)%, *P* > 0.05] showed no statistical significance at 3 months after TA-BSM (Table 3).

### Relationship of changes to biomarkers and echocardiographic parameters of LA and LV function

Table 4 shows significant associations between reduction in serum NT-proBNP level and echocardiographic parameters of LVOT peak gradient, LA end-systolic dimension (LAD), LAVI, E/A, E/e′, LAESV, LASr, LAScd and LA stiffness index (*P* < 0.05). Moreover, echocardiographic parameters of LVOT peak gradient, LAVI, E, E/A, E/e′, LAESV and LA stiffness index were significant associated with reduction in serum hs-cTnI level (*P* < 0.05).

**Table 4:** Linear regression of log2 changes in biomarkers on echocardiographic changes

	Intercept	b coefficients	95% CI	*P*-value
Log2 change in NT-proBNP, ng/l				
LVOT peak gradient, mmHg	0.314	0.013	0.007–0.020	<0.001
LAD (mm)	0.135	0.094	0.047–0.014	<0.001
LAVI (ml/m^2^)	0.227	0.057	0.040–0.074	<0.001
E (cm/s)	−0.298	0.008	−0.001–0.018	0.083
E/A	−0.257	0.721	0.159–1.282	0.012
E/e′ (average)	−0.182	0.068	0.013–0.123	0.016
TR Vmax (m/s)	−0.258	0.328	−0.091–0.746	0.123
MR area/LA area (%)	−0.104	0.013	−0.005–0.031	0.144
LAESV (ml)	0.020	0.046	0.017–0.074	0.002
LASr (%)	0.175	−0.119	−0.165–0.073	<0.001
LAScd (%)	−0.243	0.091	0.041–0.140	<0.001
LASct (%)	−0.296	0.030	−0.021–0.082	0.250
LA stiffness index	−0.104	0.790	0.355–1.226	<0.001
Log2 change in hs-cTnI (ng/l)				
LVOT peak gradient (mmHg)	0.748	0.009	0.003–0.015	0.004
LAD (mm)	0.431	0.027	−0.017–0.070	0.233
LAVI (ml/m^2^)	0.787	0.047	0.032–0.062	<0.001
E (cm/s)	0.425	0.013	0.005–0.021	0.002
E/A	0.398	0.632	0.138–1.125	0.013
E/e′ (average)	0.465	0.060	0.011–0.109	0.016
TR Vmax (m/s)	0.344	0.154	−0.217–0.525	0.412
MR area/LA area (%)	0.265	−0.001	−0.016–0.015	0.920
LAESV (ml)	0.717	0.048	0.024–0.073	<0.001
LASr (%)	0.447	−0.035	−0.079–0.010	0.127
LAScd (%)	0.317	0.021	−0.024–0.067	0.353
LASct (%)	0.329	0.015	−0.030–0.061	0.510
LA stiffness index (%^−1^)	0.579	0.822	0.448–1.195	<0.001

E/A: early mitral inflow velocity/late mitral inflow velocity; E/e′: early mitral inflow velocity/the average of septal and lateral mitral annular early diastolic velocity; E: early mitral inflow velocity; LA: left atrial; LAD: left atrial end-systolic dimension; LAESV: left atrial end-systolic volume; LAScd: left atrial conduit strain; LASct: left atrial contraction strain; LASr: left atrial reservoir strain; LAVI: left atrial maximum volume index; LVOT: left ventricular outflow tract; MR: mitral regurgitation; NT-proBNP: N-terminal pro-B-type natriuretic peptide; TR Vmax: tricuspid regurgitation maximum velocity.

## DISCUSSION

Based on the initial feasibility report of TA-BSM, this study showed early LA reverse remodelling in HOCM patients post TA-BSM. Key findings include: (i) TA-BSM effectively relieved LVOT obstruction and mitral regurgitation, significantly improving LA size and function. LAVI reduced by 76%, with a median change of 20%, and 48% of patients achieved LA reverse remodelling criteria; (ii) Diastolic function markers improved early post-procedure, while LV global longitudinal strain showed no significant change at 3-month follow-up; (iii) Indicators of LA reverse remodelling correlated with reduced serum NT-proBNP and hs-cTnI levels, suggesting improved LV relaxation and clinical status.

Compared to conventional septal myectomy, TA-BSM offers real-time echocardiography navigation for precise LV geometry assessment. This allows accurate septal resection planning and immediate reassessment of haemodynamic changes, marking an advance over traditional surgery. TA-BSM is minimally invasive without cardiopulmonary bypass, enhancing acceptability among HOCM patients [13]. In our initial study, median mechanical ventilation duration post-TA-BSM was 3.8 h [7].

It was widely accepted that HOCM entails LA enlargement and diastolic dysfunction, elevating risks for severe cardiac events. Nguyen *et al.* [14] reported early and enduring LA volume decline post traditional myectomy. Similarly, we found significant LA volume reduction in TA-BSM patients. The early decrease in LA volume was mainly attributed to relief of obstruction and improvement of mitral regurgitation [15]. Besides reduced LA volume, significant improvements in LASr, LAScd and LASct were observed over 3 months. These findings support the European Association of Cardiovascular Imaging (EACVI) consensus on heart failure imaging, emphasizing LASr for assessing LV filling pressure [16]. In this study, we provided comprehensive parametric measures confirming the early left atrial reverse remodelling after septal myectomy.

In recent decades, therapies like medical treatment, surgical myectomy and alcohol septal ablation have proven effective for HOCM symptoms and survival rates [17]. A novel small molecule, Mavacamten, improves diastolic function and promotes LA reverse remodelling in HCM patients [18]. Notably, its mechanism differs from surgical approaches—it inhibits cardiac myosin to reduce cross-bridge formation, lower LV stiffness, improve compliance and decrease filling pressure [18]. In this study, along with a notable reduction in ventricular wall thickness and mitral regurgitation after septal myectomy, there were consistent improvements in LV diastolic function and LA volume and function. Therefore, it is reasonable to assume that the early LA reverse remodelling after TA-BSM surgery is partly due to the improvement of LV diastolic function.

Three months post TA-BSM surgery, a notable reduction in LA stiffness index was observed in this study. This index reflects LA resistance to pressure and volume changes and independently affects E/e′, LA size and LV diastolic function in HCM patients [19]. It’s also predictive of cardiovascular events in diverse cardiovascular conditions [19]. The decrease signifies enhanced LA and LV diastolic function. As LA undergoes reverse remodelling, it improves blood pumping efficiency, enhancing exercise tolerance and alleviating symptoms like dyspnoea, chest pain and fatigue.

Serum biochemical markers correlated significantly with echocardiography parameters in the study, supporting TA-BSM surgery’s effectiveness in reducing LV filling pressure, improving LA size, and enhancing function. The decrease in Hs-cTnI and NT-proBNP levels observed indicates an improvement in LA reverse remodelling indices, indicating early recovery of LV diastolic function, cardiac function and clinical status.

Increased LA stiffness index and diastolic pressure correlate with poor prognosis in cardiovascular diseases [19, 20]. TA-BSM surgery potentially enhances cardiac performance and reduces complications linked to elevated LA pressures by decreasing stiffness and improving diastolic function. LA reverse remodelling is crucial in cardiovascular care, enhancing mechanical performance and overall health outlook. Lowering LA pressure mitigates risks like atrial fibrillation, stroke and heart failure. Surgical interventions that achieve substantial reverse remodelling predict better long-term outcomes [21]. Significant improvement post TA-BSM surgery indicates a favourable response and potential for improved cardiovascular health in the long run.

The absence of a control group limits definitive conclusions about whether off-pump myectomy’s reported benefits are solely due to the surgical technique or other factors. Off-pump surgery on a beating heart may reduce tissue damage and speed up recovery, potentially improving outcomes. Maintaining continuous blood flow to the heart could prevent ischaemic damage linked to cardiopulmonary bypass. Avoiding bypass might also lower inflammation risks, aiding recovery. However, without a control group, these points remain speculative, underscoring the need for rigorous randomized trials to systematically assess these potential benefits.

### Study limitations

The study’s single-arm design without a control group restricts interpretation of non-inferiority to conventional surgical septal myectomy, potentially influenced by uncontrolled factors. Future randomized controlled trials comparing off-pump to on-pump myectomy are crucial. The small sample size underscores the need for larger studies. Early-stage TA-BSM limits findings to initial follow-up; extended, large-scale trials are essential to validate long-term efficacy and safety, especially concerning LA reverse remodelling and overall outcomes.

## CONCLUSION

This pioneering study examines early LA reverse remodelling in HOCM patients after TA-BSM, highlighting improvements in LVOT and mitral regurgitation alongside significant enhancements in LA size and function during short-term follow-up. Biomarkers of myocardial wall stress showed reductions, suggesting early improvements in LV relaxation and clinical status post-surgery.

## Data Availability

The data underlying this article will be shared by the corresponding author on reasonable request.

## ABBREVIATIONS

HOCM: Hypertrophic obstructive cardiomyopathy
LA: Left atrial
LAVI: Left atrial maximum volume index
LV: Left ventricular
LVOT: Left ventricular outflow tract
TA-BSM: Transapical beating-heart septal myectomy
